# Role of Polarons in Single-Atom Catalysts: Case Study of Me_1_ [Au_1_, Pt_1,_ and Rh_1_] on TiO_2_(110)

**DOI:** 10.1007/s11244-022-01651-0

**Published:** 2022-06-27

**Authors:** Panukorn Sombut, Lena Puntscher, Marlene Atzmueller, Zdenek Jakub, Michele Reticcioli, Matthias Meier, Gareth S. Parkinson, Cesare Franchini

**Affiliations:** 1 Institute of Applied Physics, TU Wien, 1040 Vienna, Austria; 2 Faculty of Physics, Center for Computational Materials Science, University of Vienna, 1090 Vienna, Austria; 3 Alma Mater Studiorum, Università di Bologna, 40127 Bologna, Italy

**Keywords:** Single-atom catalysis, Density functional theory, Polarons, TiO_2_(110) surface, Scanning probe microscope

## Abstract

The local environment of metal-oxide supported single-atom catalysts plays a decisive role in the surface reactivity and related catalytic properties. The study of such systems is complicated by the presence of point defects on the surface, which are often associated with the localization of excess charge in the form of polarons. This can affect the stability, the electronic configuration, and the local geometry of the adsorbed adatoms. In this work, through the use of density functional theory and surface-sensitive experiments, we study the adsorption of Rh_1_, Pt_1_, and Au_1_ metals on the reduced TiO_2_(110) surface, a prototypical polaronic material. A systematic analysis of the adsorption configurations and oxidation states of the adsorbed metals reveals different types of couplings between adsorbates and polarons. As confirmed by scanning tunneling microscopy measurements, the favored Pt_1_ and Au_1_ adsorption at oxygen vacancy sites is associated with a strong electronic charge transfer from polaronic states to adatom orbitals, which results in a reduction of the adsorbed metal. In contrast, the Rh_1_ adatoms interact weakly with the excess charge, which leaves the polarons largely unaffected. Our results show that an accurate understanding of the properties of single-atom catalysts on oxide surfaces requires a careful account of the interplay between adatoms, vacancy sites, and polarons.

**Supplementary Information:**

The online version contains supplementary material available at 10.1007/s11244-022-01651-0.

## Introduction

Due to their particular local environment, single-atom catalysts (SACs) represent a new frontier in heterogeneous catalysis, resulting in a unique electronic structure in comparison with supported nanoparticle catalysts [1–7]. Metal atoms adsorbed on solid supports and their resulting catalytic properties combine the advantages of homogeneous catalysts (high activity and selectivity) and heterogeneous catalysts (stable and easy to separate), while minimizing the amount of precious metal used in heterogeneous catalysis [3, 8]. Therefore, SACs are expected to bridge the gap between heterogeneous and homogeneous catalysts. However, the tendency of isolated atoms to aggregate into small clusters due to their relatively high surface energy is problematic. A strong covalent metal-support interaction is capable of stabilizing SACs [9]. However, adsorption of SACs in atomic defects on the substrate surface is the most effective way to avoid clustering and stabilize isolated metal atoms on the support [10, 11], expanding the applicability and efficiency of single-atom catalysis.

Nonetheless, such defective surfaces can affect the properties of adsorbed adatoms, whether within or outside the defect itself, and must be carefully investigated. Further, the presence of point defects on transition-metal oxide surfaces can inject excess electrons which can locally couple with ionic vibrations and form small polarons [12]. Adsorbate/oxide-surface interactions are known to be significantly affected by defects and their associated polarons [13–15], but their effect on catalysis has been rarely considered [16, 17]. For instance, the properties of metal atom (Me_1_) species on reduced rutile TiO_2_(110) surface, a prototypical polaronic system, has been extensively studied as a SAC [18–23], but the potential effects of polarons are generally neglected. On the reduced TiO_2_(110) surface, polarons tend to localize at a 6-fold coordinated Ti atom (Ti_6c_) in the subsurface layer in the vicinity of the 2-fold coordinated oxygen vacancy (V_O2c_) site, reducing Ti^4+^ to Ti^3+^ ions [24]. At elevated temperatures, polaron diffusion between subsurface and surface layers may occur, altering the properties and nature of the polaronic state [24–26], and potentially affecting the stability and properties of the adatoms as well.

In recent years, computational studies have become a powerful tool to accurately describe catalytic reactions at the atomic scale in heterogeneous catalysis [27–29]. In particular, first-principles calculations, within the density functional theory (DFT) framework, have revealed several useful insights into the nature of active sites and the reaction mechanisms in the SAC models [30]. Furthermore, the advances brought about by DFT studies facilitate the interpretation of experimental measurements, and might propose specific substrate materials and metal atoms as optimal candidates for efficient SAC processes.

The purpose of this study is to investigate the effect of polarons on the stability and properties of single-metal atom catalysts. We consider the adsorption of Rh_1_, Pt_1_, and Au_1_ transition metals on the reduced rutile TiO_2_(110) surface. To investigate the interplay between electron polarons, oxygen vacancies, and the adatom on the TiO_2_(110) surface, we performed DFT + U calculations and compared the results with experimental data taken from existing literature (Au_1_ [31–35]) as well as new scanning tunneling microscopy data for the Pt_1_ and Rh_1_ systems. Accordingly, we confirm that charge transfer occurs for Pt_1_ and Au_1_ adatoms located in O vacancies (V_O_), making them the preferred adsorption configurations, by carefully studying the most stable adsorption sites, the appropriate oxidation states, and the interactions among adatoms, O vacancies, and polarons. A low diffusion barrier on the surface of TiO_2_(110) allows Pt_1_ adatoms to reach O vacancies when dosed in low amount at room temperature. Au_1_ adatoms exhibit the same behavior but at a lower temperature with an even lower diffusion barrier. Rh_1_ is found to have no preference for such defects, leaving the polarons essentially unaffected in the subsurface. Our results show that the properties of single-atom catalysts on metal-oxide surfaces can be accurately described only by carefully considering the interaction with point defects and polarons, as well as the reduction of the adsorbed metals.

## Methods

### Computational Methods

**Fig. 1:**
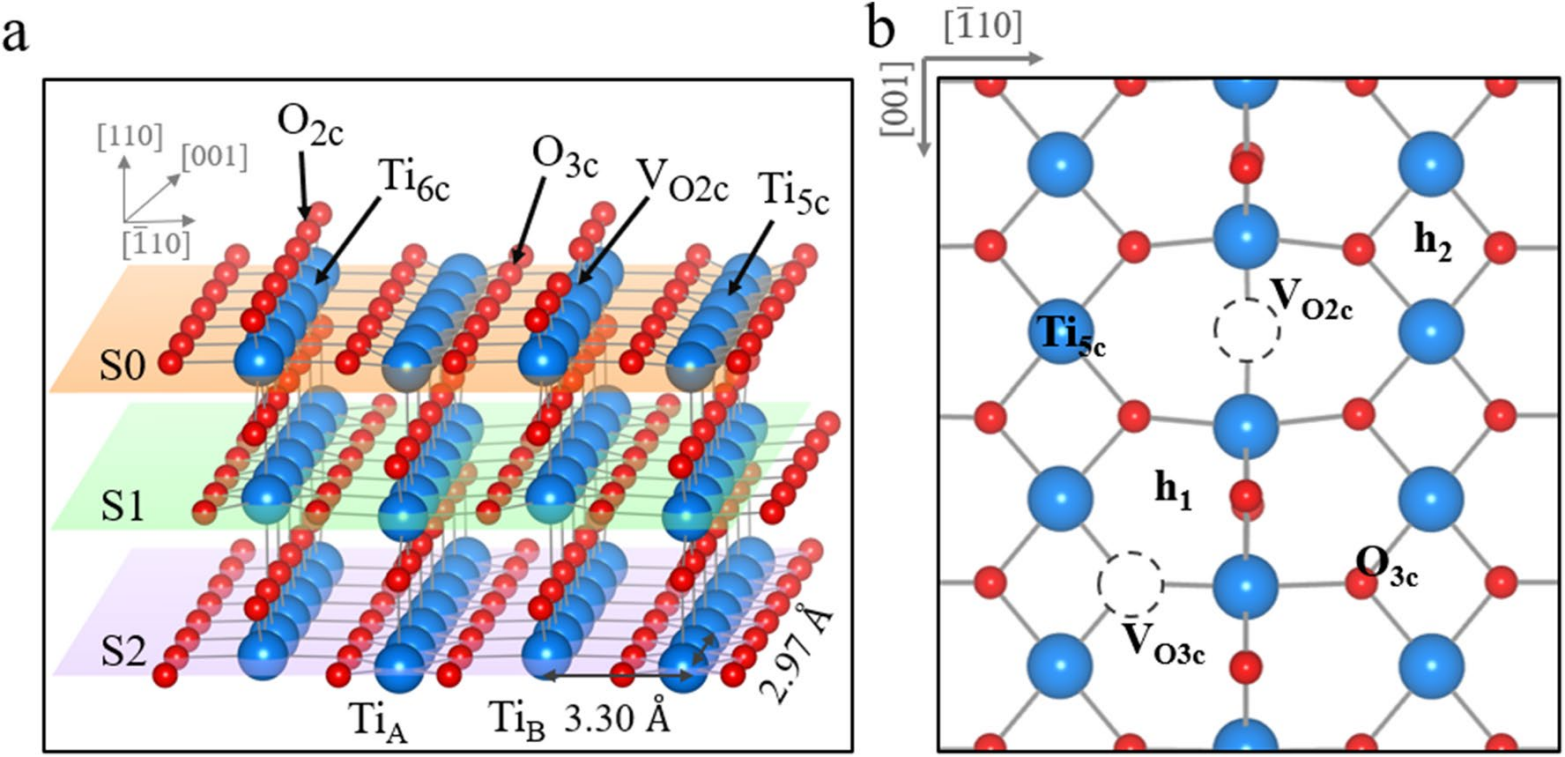
Structure of the TiO_2_(110) surface, Ti atom, O atom, and oxygen vacancy are depicted in big blue sphere, small red sphere and dashed-circle, respectively. **a** Side view of a 6 × 2 unit cell and **b** top view with possible adsorption sites: a 2-fold oxygen vacancy (V_O2c_), hollow_1_ (h_1_), hollow_2_ (h_2_), a 5-fold titanium atom (Ti_5c_), a 3-fold oxygen atom (O_3c_) and a 3-fold oxygen vacancy (V_O3c_)

All calculations were performed by using the Vienna ab initio simulation package (VASP) [36, 37]. The projector augmented wave method [38, 39] was used for the electron and ion interaction, with the plane-wave basis set cutoff energy set to 400 eV, optimized to include van der Waals interactions as proposed by Dion et al. [40] with the optimized functional (optPBE-DF) [41, 42]. However, DFT calculations have known drawbacks when dealing with electron localization effects [43]. Therefore, it is preferable to use first-principle schemes that account for the localized charge, such as the DFT + U method used here [44, 45]: we dressed the d orbitals of the Ti atoms with an effective on-site Coulomb repulsion term (U_eff_ of 3.9 eV) [46], previously determined by constrained-random-phase-approximation calculations in bulk rutile [25]. The unreconstructed rutile surface was modeled using an asymmetric slab containing five TiO_2_ tri-layers in a large two-dimensional 6 × 2 unit cell and including a vacuum space region greater than 12 Å along the z-axis. The top three tri-layers were allowed to relax, while the bottom two tri-layers were kept fixed at their bulk positions. An alternative slab model in which the broken bonds at the bottom layer were saturated by pseudo-hydrogen atoms did not affect our conclusions regarding the adsorption energies and polaron stabilities. The convergence is achieved when the electronic energy step of 10^− 5^ eV is obtained and forces acting on ions become smaller than 0.01 eV/Å. The adsorption energies were computed according to the formula:

$$E_{{{\text{ads}}}} = E_{{{\text{TiO}}_{2} \left( {110} \right) + {\text{adatom}}}} - \left( {E_{{{\text{TiO}}_{2} \left( {110} \right)}} + E_{{{\text{adatom}}}} } \right)$$

where $E_{{{\text{TiO}}_{2} \left( {110} \right) + {\text{adatom}}}}$ is the total energy of the TiO_2_(110) surface with the adsorbed adatom, $E_{{{\text{TiO}}_{2} \left( {110} \right)}}$ is the total energy of the clean TiO_2_(110) surface with an oxygen vacancy and the most stable polaronic configuration [47, 48]. The $E_{{{\text{adatom}}}}$ represents the energy of the atom in the gas phase. The surface slab is displayed in Fig. 1a.

The diffusion barriers of an adatom on the reduced TiO_2_(110) surface were evaluated using the climbing image nudged elastic band (CI-NEB) method [49, 50] with three interpolated images. As initial and end states we carefully selected solutions including adatoms in the same oxidation state.

**Fig. 2:**
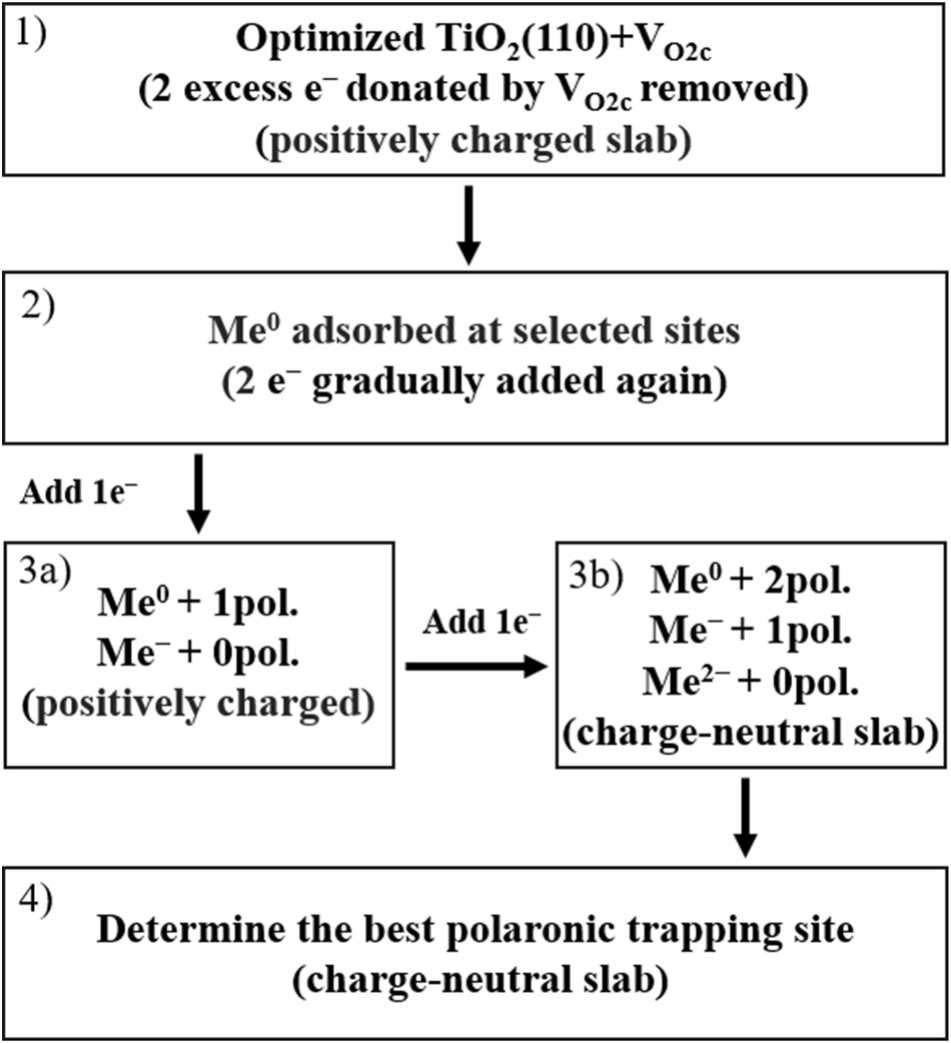
A flow diagram shows the strategy to obtain the proper charge states of metal atoms adsorbed on different adsorption sites on a charge-neutral reduced TiO_2_(110) surface with one V_O2c_

In order to inspect the different charge states of the metal atoms on different adsorption sites and the effect of the presence of polarons, we adopted the following strategy (see Fig. 2).

1. To inhibit polaron formation, we manually removed the two excess electrons (changed the flag NELECT in VASP), resulting in a positively charged slab. By doing so, we remove polaron-related energy contributions, such as polaron formation energies or polaron-Me interactions, which allows for a more systematic and controlled approach to study Me single-atom sites.
2. Adsorption sites were selected based on existing literature [22, 23, 31, 33–35, 51–54] (see Fig. 1). The Me-single atoms are adsorbed at the different adsorption sites as neutral species Me^0^ on the previously prepared surface (i.e., without polarons to interact with). The obtained Me^0^ adsorption energies can be considered initial reference values.
3. To account for the variation in relative adsorption strengths due to the oxidation state of the Me_1_ atom, charge is slowly re-added to the system. By adding one and then two electrons, the single atom is able to achieve its most favorable oxidation state Me^0^, Me^−^ or Me^2−^:
  - Adding one electron: The Me_1_ can either retain its additional charge displaying the Me^−^ oxidation state or become neutral again by moving the electron to the support where it forms a polaron [Me^0^ + 1pol.] vs. [Me^−^ + 0pol.].
  - Adding two electrons: Me^0^, Me^−^ and Me^2−^ become available oxidation states as a result of adding one more electron. Three different configurations can be obtained: [Me^0^ + 2pol.] vs. [Me^−^ + 1pol.] vs. [Me^2−^ + 0pol.].
  As a result of step 3(b), we have retrieved the initial charge neutrality, artificially altered in step 1. At this step, we have ensured that polarons are located at the lowest energy polaronic sites (in S1 in the vicinity of V_O2c_) as they would in the pristine slab. Therefore, the remaining optimizations left are the interactions of polarons, if present, with the Me single atom, which is covered in step 4. Clearly, if the Me^2−^ state results as the preferred one, no polarons are formed in the slab.
4. When polarons are formed (Me^0^ and Me^−^), we inspect the energy stability with respect to different polaronic trapping sites (in S0 and S1). In this way we consider the effect of the spatial polaron-V_O2c_ and polaron-Me separation. It is known that the adatom adsorption energy is affected by the polaron orbital topology, degree of localization, and associated local structural distortions [24]. By comparing the final total energies of these charge-neutral slabs we determine the final ground state structure with the most favorable Me oxidation state previously determined and preferable polaron configurations.

The actual Me oxidation state is determined by counting the number of Ti^3+^ in the charge-neutral slab (i.e. the number of polarons in the slab), the local spin moment at the Me single atom itself, and integrated pDOS.

To selectively control the charge localization in different Ti^3+^ sites, we used the occupation matrix control tool [55], which consists of an initial constrained calculation (with an input occupation matrix kept fixed during the calculation) followed by an unconstrained calculation.

### Experimental Details

Low temperature scanning tunneling microscopy (STM) was performed in a two-vessel UHV chamber consisting of a preparation chamber (p < 1 × 10^− 10^ mbar) and an analysis chamber (p < 2 × 10^− 11^ mbar). The preparation chamber is equipped with a commercial X-ray photoelectron spectroscopy (XPS). The analysis chamber is equipped with an Omicron LT-STM with a Qplus sensor and an in-vacuum preamplifier [56]. Room-temperature STM was performed in a second two-vessel UHV chamber consisting of a preparation chamber (p < 1 × 10^− 10^ mbar) and an analysis chamber (p < 5 × 10^− 11^ mbar). The analysis chamber is equipped with a nonmonochromatic Al Kα X-ray source (VG), a SPECS Phoibos 100 analyzer for XPS, and an Omicron µ-STM. STM in both UHV chambers was conducted in constant current mode with an electrochemically etched W tip on synthetic TiO_2_(110) single crystals (from CrysTec GmbH) prepared in UHV by sputtering (1 kV, Ar+, 15 min) and annealing (20 min, 700 °C). Rh and Pt were deposited using an e-beam evaporator (FOCUS), with the flux calibrated using a temperature-stabilized quartz microbalance (QCM). The STM images were corrected of distortion and creep of the piezo scanner as described in ref [57].

## Results

The rutile TiO_2_(110) surface is one of the most intensively studied metal-oxide surfaces [58–60]. This surface layer consists of bridging oxygen rows (O_2C_) and 5-fold coordinated titanium (Ti_5c_) rows (see the structural model in Fig. 1). Bridging oxygen vacancies (V_O2c_) can be easily created in UHV conditions by sputtering and annealing. Each V_O2c_ defect donates two excess electrons to the surface, which are trapped in Ti sites forming small polarons, clearly identified by sharp in-gap peaks [12, 48, 61–63]. We aim to elucidate the impact of polarons on the stability and properties of single-metal atoms (Pt_1_, Au_1_, and Rh_1_) adsorbed on the rutile TiO_2_(110) surface. The adsorption sites are labeled in Fig. 1b.

### Pt_1_ on the TiO_2_(110) Surface

**Fig. 3:**
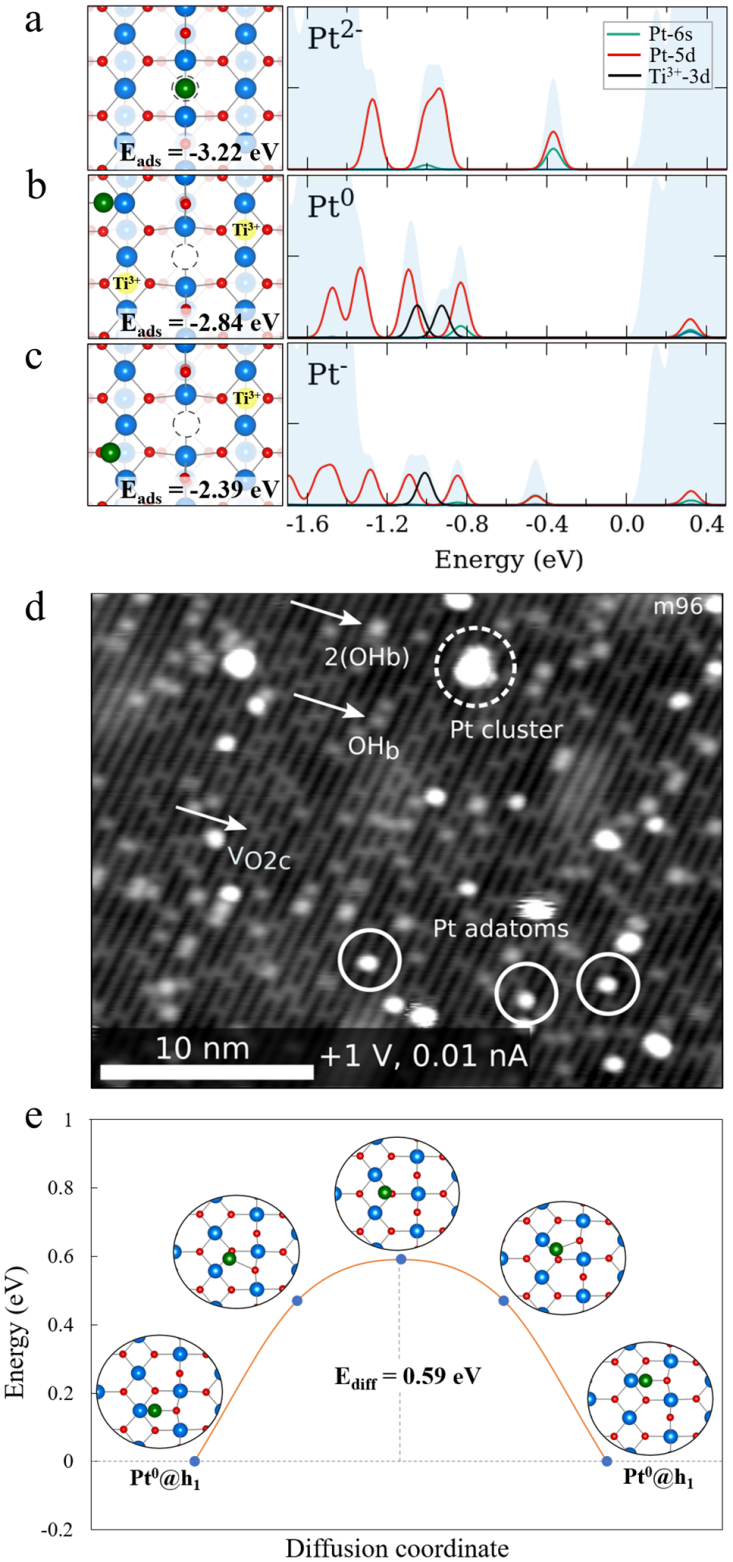
Minimum energy configuration of possible adsorption sites of Pt_1_ on the TiO_2_(110) surface. **a** Pt_1_@V_O2c_ without polarons resulting in a Pt^2−^ configuration. **b** Pt_1_ in h_1_ with two polarons resulting in a Pt^0^ configuration. **c** Pt_1_ atop O_3C_ atom with one polaron resulting in a Pt^−^ configuration, where O, Ti^4+^, Ti^3+^(polaron), Pt_1_ are small red, big blue, big yellow, big green spheres and V_O_ is a dashed-circle, respectively. Each configuration is aligned with its respective DOS panels, where the total DOS, pDOS of Pt (5d), pDOS of Pt (6s), and pDOS of Ti^3+^(3d, polarons) are filled light blue, red line, green line, and black line, respectively. **d** Empty-state room temperature STM images of 0.007 ML Pt on the reduced rutile TiO_2_(110) surface deposited at room temperature, with surface oxygen vacancies (V_O2c_), OH groups (OH_b_), pairs of OH groups (2(OH_b_)), Pt adatoms (circle) and Pt clusters (dashed circle) labelled in the image. **e** Diffusion path of Pt_1_ adatom on TiO_2_(110) from one hollow site to the next in the neighboring unit cell without the perturbation from any V_O_

In order to investigate the adsorption of Pt_1_ on TiO_2_(110), we considered possible adsorption sites as reported in the available literature [21, 22, 51, 52] but including the presence of polarons. Figure 3a-c shows the three most stable adsorption sites and their corresponding calculated projected DOS (pDOS) as well as their oxidation states, taking polaronic effects into account. Pt_1_ adsorbed at a bridging oxygen vacancy is the most stable adsorption site (E_ads_ = − 3.22 eV). The Pt_1_ atom in this configuration shows an oxidation state of Pt^2−^ due to the charge transfer of two excess electrons from the reduced surface to the Pt_1_ adatom, meaning that it is more favorable to transfer the electrons to Pt_1_ rather than using the excess charge to form polarons. The calculated pDOS indeed shows no in-gap Ti polaronic peaks. We note that Pt^−^ in this configuration is less stable than Pt^2−^ by 0.39 eV (Fig. S6). Pt_1_ at the h_1_ site is next in energy (E_ads_ = − 2.84 eV), with Pt^0^ being the most stable oxidation state. In this case, the two excess electrons prefer to be trapped in polaronic sites, and no evident net charge transfer to Pt_1_ occurs. The pDOS also shows two polaronic in-gap peaks for two Ti^3+^ sites, similar to the clean surface (Fig. S1). When polaron formation is maintained, as in the case of Pt^0^, polarons prefer to be trapped near the oxygen vacancy in the S1 layer [24]. The last possible adsorption site with comparatively large adsorption energy is Pt_1_ adsorbed atop a 3-fold coordinated oxygen atom on the basal plane. Pt^−^ is the most stable oxidation state at this site, with one polaron again preferably located in the S1 layer near the oxygen vacancy. Overall, the polarons prefer to be located in the S1 layer in the proximity of the V_O2c_ and maximize their distance with the adsorbed Pt_1_ adatom due to the repulsive interaction between the negatively charged polaron and the adsorbed metal atom. A variation of the order of 300 meV in the adatom adsorption energy can be seen depending on polaron position and its distance to the adatom (Fig. S2).

The room-temperature STM image (Fig. 3d) shows Pt_1_ adsorbed on the reduced rutile TiO_2_(110) surface. The TiO_2_(110) surface is characterized in STM by bright rows of 5-fold coordinated Ti^4+^ alternating with dark rows of 2-fold bridging O^2−^, which run along the [001] direction [60]. Small protrusions over the dark rows can be assigned to oxygen vacancies V_O2c_, and brighter features on the dark rows can be assigned to bridging OH [64] and pairs of bridging OH. These originate from water dissociation at the V_O2c_ [65–67]. Pt adatoms can be stabilized at low coverage, but clusters dominate the higher the coverage. The Pt adatoms adsorb in the 2-fold oxygen vacancies (marked in circle), which can be identified directly when rare events of adatoms diffusion occur. In this case, the Pt adatom diffuses from one V_O_ to another (Fig. S7). Both the initial and final vacancies are imaged in STM. Dosing water or oxygen gas (O_2_) at room temperature (2 L; 100 s 2 × 10^− 8^ mbar) prior to the Pt deposition leads to the reparation of the V_O_ site [66–68]. In such an experiment, only Pt clusters are observed (as seen in Fig. S8). These results show the direct correlation between oxygen vacancies and the Pt adatoms stabilization.

In order to account for dynamical effects involving diffusion of the metal species, we calculated the diffusion barrier of the adsorbed Pt_1_ along the [001] direction. The calculated energy barrier is low (0.59 eV, see Fig. 3e) so that Pt_1_ can diffuse already at room temperature and reach the best adsorption site (at the oxygen vacancies). The calculated results are therefore in-line with our room-temperature STM images, where only Pt_1_ at oxygen vacancies have been observed on the reduced TiO_2_(110) surface at low coverage. At lower dosing temperature, possible metastable adsorption configurations outside the V_O2c_ can exist.

### Au_1_ on the TiO_2_(110) Surface

**Fig. 4:**
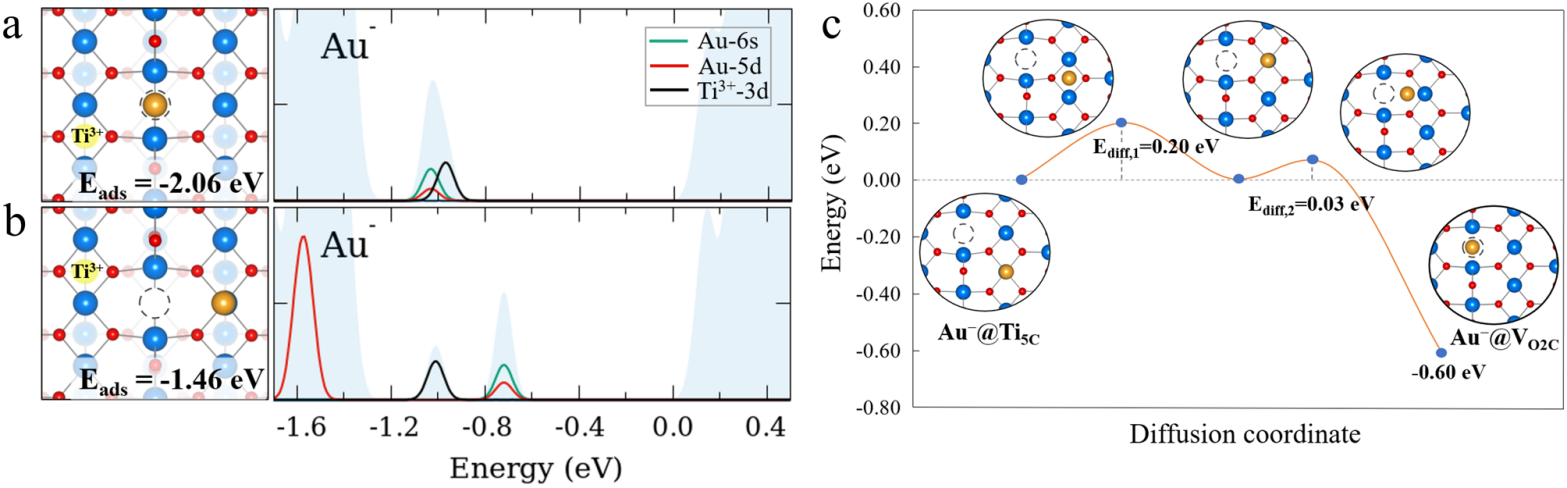
Minimum energy configuration of possible adsorption sites of Au_1_ on the TiO_2_(110) surface. **a** Au_1_@V_O2c_ with one polaron resulting in a Au^−^ configuration. **b** Au_1_ atop Ti_5c_ atom with one polaron resulting in a Au^−^ configuration. Each configuration is aligned with its respective DOS panels, where the total DOS, pDOS of Au (5d), pDOS of Au (6s), and pDOS of Ti^3+^(3d, polarons) are filled light blue, red line, green line, and black line, respectively. **c** Diffusion path of Au_1_ atom on TiO_2_(110) from atop Ti_5c_ to the V_O2c_ site, where O, Ti^4+^, Ti^3+^(polaron) and Au_1_ are small red, big blue, big yellow and big yellow-brown spheres, and V_O_ is a dashed-circle, respectively

Au_1_ adsorbed on TiO_2_(110) exhibits a similar structure than Pt_1_, as shown in Fig. 4. The most stable adsorption site for Au_1_ is located at V_O2c_ (E_ads_ = − 2.06 eV). Au_1_ located at this site becomes negatively charged with an oxidation state of Au^−^. Polaronic configurations are most favorable when the remaining polaron forms in the S1 layer close to the V_O2c_ (Fig. 4a). We also considered the on-top 5-fold Ti atom adsorption site, as it was previously considered in other theoretical works [31, 32, 69]. The most stable valence state of Au_1_ at this site is Au^−^ with one polaron remaining in the S1 layer close to V_O2c,_ as shown in Fig. 4b (E_ads_ = − 1.46 eV). The calculated pDOS in both cases shows that the valence d and s states are filled, with one characteristic in-gap polaronic peak. Similarly to Pt_1_, the different polaronic configurations can modify the adsorption energy up to 300 meV (Figs. S3, S4).

The diffusion barrier of Au_1_ on the TiO_2_(110) from the 5-fold Ti atom to the next 5-fold Ti atom along [001] direction is low (0.2 eV), and even lower is the corresponding barrier for diffusing from the 5-fold Ti atom to the oxygen vacancy which is almost barrierless, indicating a facile diffusion (Fig. 4c). Based on our results, we can conclude that at very low Au coverage, all Au_1_ adatoms will be trapped in the oxygen vacancies on the surface. This result agrees with the room temperature STM measurement at low coverage [35] and with the observation of the nucleation of gold clusters at oxygen vacancy sites at high coverage [31, 32].

### Rh_1_ on the TiO_2_(110) Surface

**Fig. 5:**
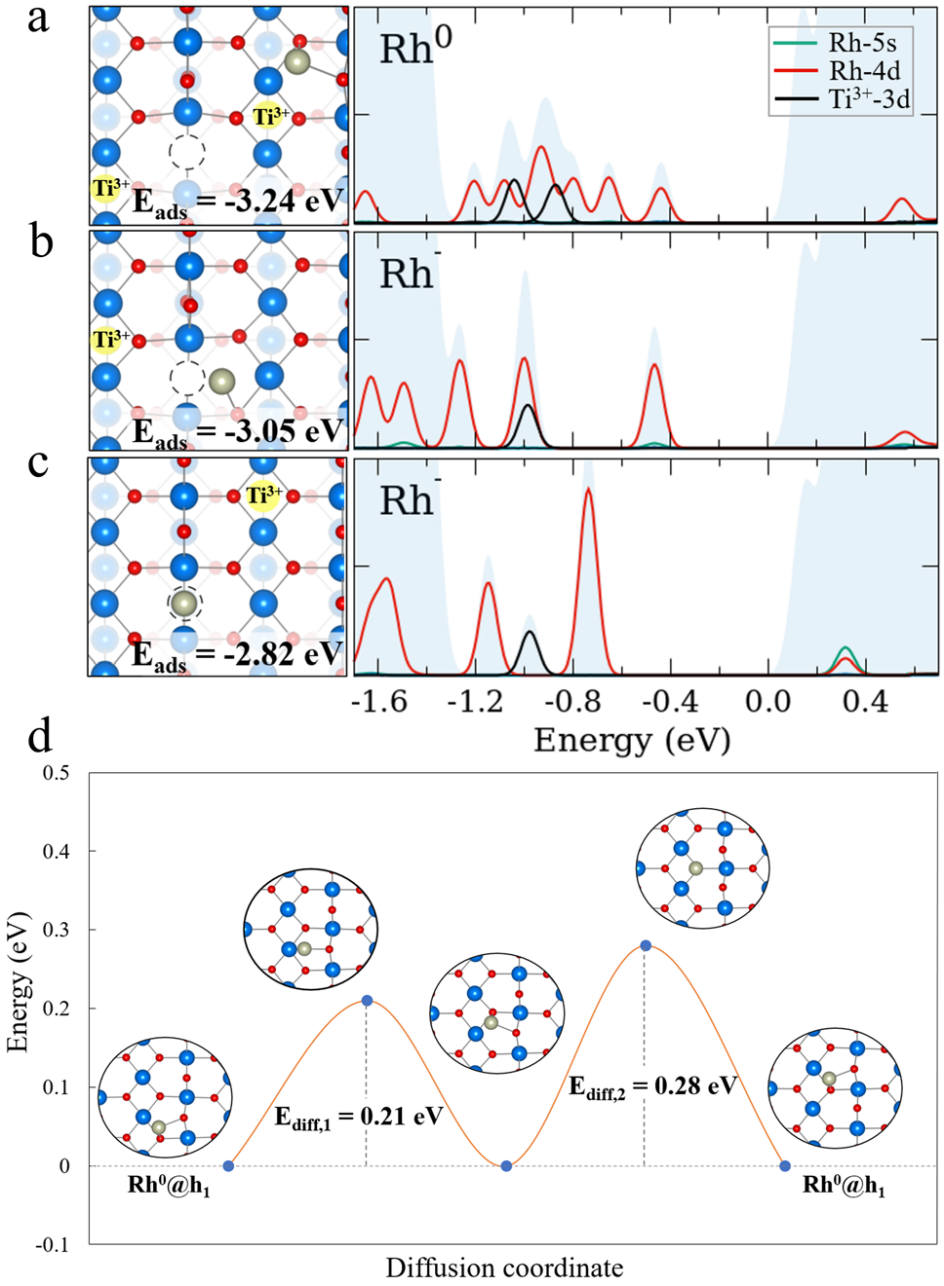
Minimum energy configuration of possible adsorption sites of Rh_1_ on the TiO_2_(110) surface. **a** Rh_1_ in h_1_ with two polarons resulting in a Rh^0^ configuration. **b** Rh_1_ in h_1_ near the V_O2c_ with one polaron resulting in a Rh^−^ configuration. **c** Rh_1_@V_O2c_ with one polaron resulting in a Rh^−^ configuration. Each configuration is aligned with its respective DOS panels, where the total DOS, pDOS of Rh (4d), pDOS of Rh (5s), and pDOS of Ti^3+^(3d, polarons) are filled light blue, red line, green line, and black line, respectively. **d** Diffusion path of Rh_1_ atom on TiO_2_(110) from the h_1_ site to the next h_1_ site, where O, Ti^4+^, Ti^3+^ and Rh_1_ are small red, big blue, big yellow and big silver spheres, and V_O_ is a dashed-circle, respectively

We studied the adsorption of Rh_1_ adatoms on TiO_2_(110) by combining DFT + U calculations with XPS and STM experiments. The adsorption sites with their respective adsorption energy are summarized in Fig. 5. Interestingly, Rh_1_ adsorbed at V_O2c_ (Fig. 5c) is not the preferential configuration (E_ads_ = − 2.82 eV). We assign a Rh^−^ state to this configuration, due to the presence of an in-gap polaronic peak from one Ti^3+^ atom. Two configurations at hollow sites are shown in Fig. 5a and b. The oxidation state of the Rh_1_ adatom at hollow sites depends on the nearest oxygen atoms binding to it. For instance, the relaxed structures show that Rh^0^ binds to the O_3c_ and O_2c_ atoms (E_ads_ = − 3.24 eV), whereas the Rh^−^ at the hollow site near V_O2c_ binds to only the O_3c_ atom (E_ads_ = − 3.05 eV). A charge transfer occurs for Rh_1_ near the V_O2c_ where one excess electron transfers to Rh_1_, leaving the second excess electron to form a polaron in the S1 layer near V_O2c_. The pDOS also shows that there is one characteristic in-gap polaronic peak. The Rh_1_ at a hollow site that is distant from V_O2c_ is assigned to Rh^0^ and has two remaining polarons in a preferential S1 configuration. The calculated pDOS shows the two in-gap polaronic peaks from Ti^3+^ atoms. Polarons reside in the S1 layer minimizing the distance from V_O2c_ while maximizing the distance from the Rh_1_. However, the adsorption energies of Rh^0^ and Rh^−^ are almost degenerate.

The adsorption of an adatom at the 3-fold oxygen vacancy (V_O3c_) has been proposed to rationalize the result from scanning transmission electron microscopy (STEM) experiments and DFT calculations for Pt_1_ and Rh_1_ [22, 23]. Other works argued that this adsorption does not exist on TiO_2_(110) due to the energetically unfavorable formation of the 3-fold oxygen vacancy (V_O3c_) on the bare TiO_2_(110) surface [35, 52]. Our calculations also show that the formation of V_O2c_ defects is much more favorable than the V_O3c_ vacancy by 1.39 eV on the pristine surface. Figure S5 shows, however, that when Rh_1_ is present and adsorbs at a V_O3c_, it becomes the most thermodynamically stable adsorption site (E_ads_ = − 3.42 eV) with respect to the V_2Oc_ formation.

**Fig. 6:**
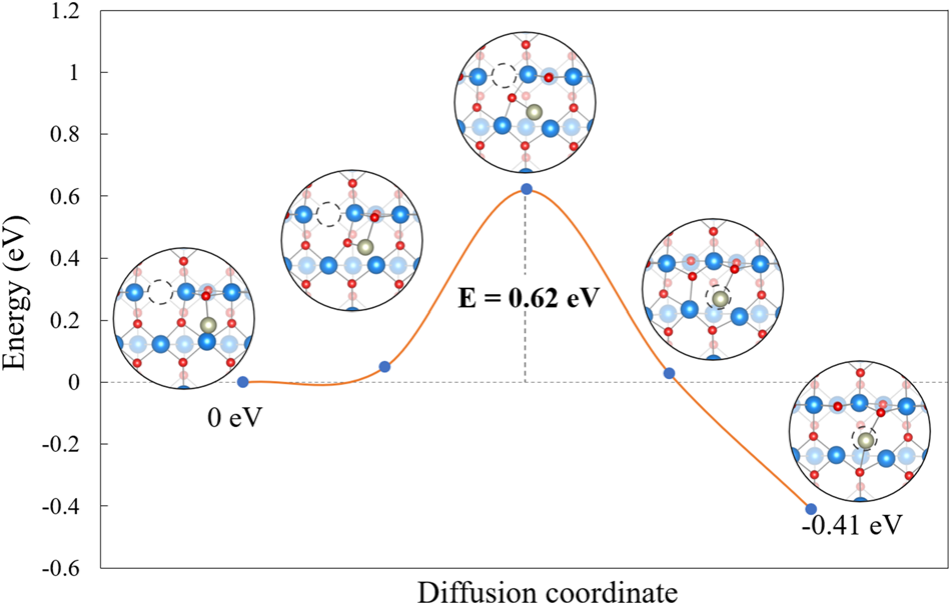
Energy profile of V_O_ migration between the initial state (Rh^−^ at hollow site) and the final state (Rh^−^ at 3-fold V_O_). In order to match the oxidation state of the final state in the NEB, the initial state is set to − 1 instead of 0, giving an offset of ~ 0.2 eV

Despite the unfavorable 3-fold oxygen vacancy formation energy found for the pristine surface, the presence of adatoms can alter the energetic cost of creating different vacancies other than V_O2c_ and therefore should not be excluded. While Rh_1_ in V_O3c_ is the most stable configuration found, the question arises whether it can be reached or not, as the as-prepared surface does not exhibit such defects prior to the deposition of Rh. We calculated the oxygen migration from V_O2c_ to V_O3c_ with the presence of Rh_1_ (Fig. 6), ignoring the presence of polarons and changes in the oxidation state of Rh. A barrier of 0.62 eV is obtained, significantly higher than the diffusion of Rh (0.28 eV, Fig. 5d) on the bare surface, suggesting that prior to the formation of in V_O3c_ adsorbed Rh, the adatoms would sinter into clusters (assuming these would be favorable in energy).

**Fig. 7:**
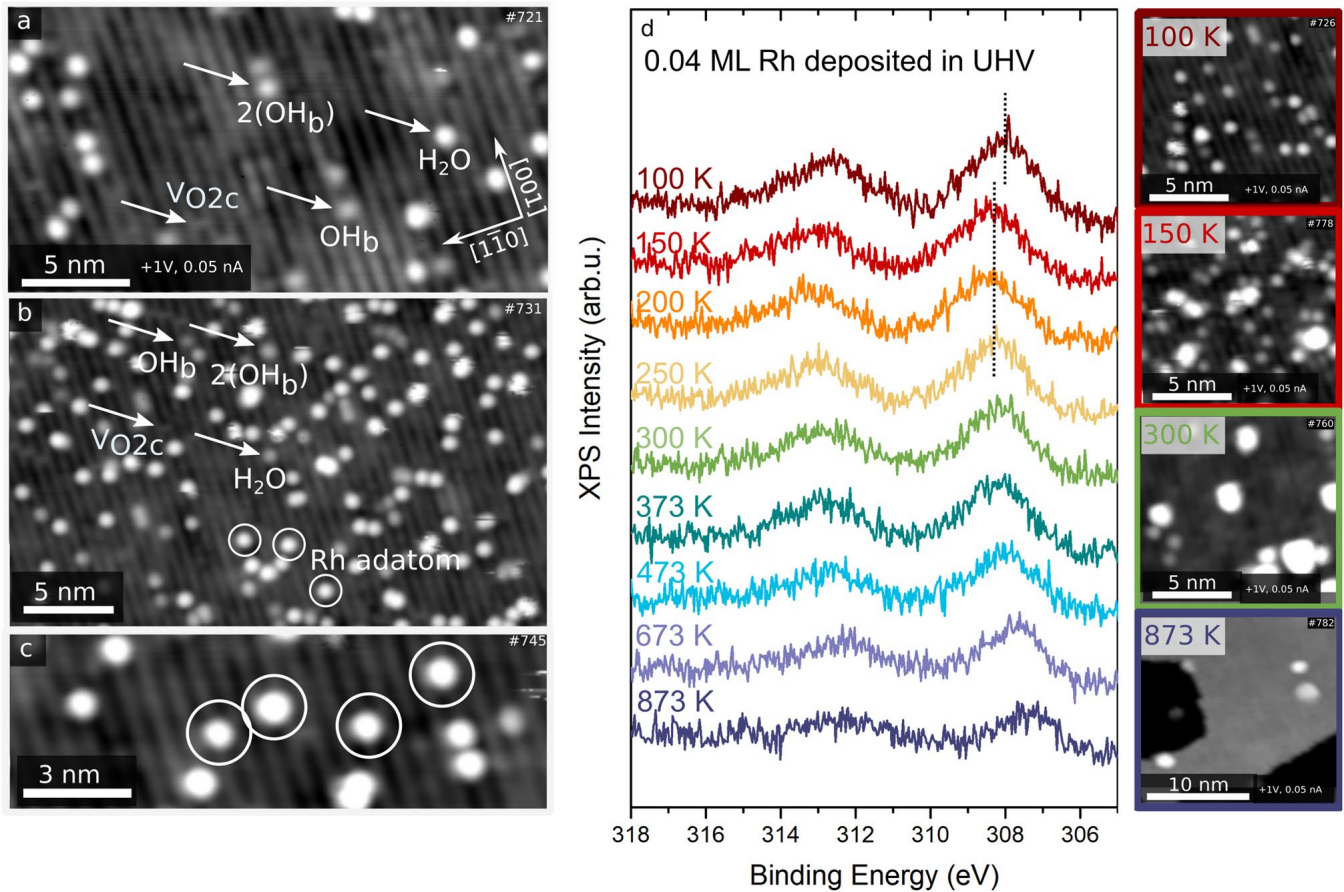
Experimental investigation of 0.04 ML Rh adsorbed on TiO_2_(110). **a** STM image (acquired at 78 K) of the clean TiO_2_(110) surface. Surface oxygen vacancies (V_O2c_), OH groups (OH_b_), pairs of OH groups (2(OH_b_)), and adsorbed water (H_2_O) molecules can be seen. **b** and **c** Following the evaporation of 0.04 ML Rh at 100 K, Rh adatoms (circle) can be seen. **d** XPS spectra and STM images of the Rh 3d peak after gradually increasing the temperature

Figure 7a–c shows STM images of TiO_2_(110) before (a) and after deposition of 0.04 ML of Rh (b) and (c) at 100 K, where 1 ML corresponds to 1 Rh atom per surface unit cell. The images were acquired using liquid nitrogen as the cryogen for the LT-STM, giving the sample a temperature of 78 K. Features on the bright Ti^4+^ rows (Fig. 7a, b) with an apparent height of 90–100 pm can be assigned to molecular water adsorbed to Ti^4+^ [70]. Rh adatoms (marked in circles) are located atop the bright Ti^4+^ rows but slightly tilted towards the dark rows (bridging O^2−^). All Rh adatoms adsorb at the same site but vary in apparent height between 140 and 180 pm. This behavior differs from the behavior of Au and Pt adatoms, which preferentially adsorb in the V_O2c_ of the TiO_2_(110) surface [35]. Our data suggest no preferential interaction between Rh and V_O2c_ as the density of the visible V_O2c_ is identical before and after Rh deposition. This result agrees with our DFT calculations, where the best adsorption of Rh_1_ is located at the hollow between the bridging oxygen row and the Ti row.

Figure 7d shows XPS spectra and the corresponding STM images of the Rh 3d peak of the as-deposited Rh at 100 K and after subsequent annealing to 873 K. Between 100 K and 150 K the Rh 3d peak shifts to higher binding energy. This core-level shift could be related to a final state effect linked to the small size of the clusters, which appear in STM at 150 K. A similar effect can be recognised when comparing Au_1_ and Au_3_ to Au nanoparticles on the reduced TiO_2_(110) surface. The Au 4f binding energies of the Au_1_ and Au_3_ are similar to those at a higher coverage of Au. This effect occurs from a cancellation of initial and final state effects for Au_1_ and Au_3_. By increasing the coverage, the binding energy first decreases and finally increases [71]. Between 150 and 250 K the peak stays the same but when heating the sample to temperatures above 300 K the peak shifts gradually to lower binding energy. This is related to the formation of bigger clusters with increasing temperature. These experimental observations, where Rh is seen to cluster after heating just to 150 K (Fig. 7d) agree with our previous calculated diffusion barriers and preference to sintering.

## Discussion and Conclusions

The presence of localized charges on surfaces in form of small polarons, unavoidably impacts adsorption and reaction processes and surface dynamics. Previous studies have shown that adsorbed CO exhibit different coupling regime (from attractive to repulsive) depending on the position and density of small polarons, revealing a polaron-mediated correlation between CO adsorption energy and reduction state of the sample [13]. Substantial polaron-charge transfer has been found in O_2_ adsorption, leading to the formation of superoxo and peroxo species [14]. Here, we have shown that single-atom adsorption is also strongly coupled with polaron-charge transfer effect, which affects the adsorption energy and, importantly, the oxidation state of the metal atoms. For example, a Pt^2−^ binds stronger than a Pt^−^ at the V_O_ site by 0.39 eV. This also implies that the reduction level and associated polaron density does affect the stability of the adsorbed metal atoms, and therefore its surface dynamics, but also its reactivity (i.e. charge transfer between the single-atom catalysts and adsorbed molecules).

For our model system, we found that Pt_1_ and Au_1_ adatoms have low diffusion barriers and preferably adsorb in V_O2c_. At higher dosing amounts, clusters can be observed. Rather than forming polarons, the V_O2c_ excess electrons are transferred to the metal atoms, altering their electronic structure by filling their valence states and result in modifying their relative stabilities. The fact that negatively Pt_1_ and Au_1_ adatoms form cluster means that during the diffusion process there must be a substantial charge transfer from the Pt_1_ and Au_1_ adatoms to the substrate [72, 73]. Rh_1_ adatoms, however show no preference for the V_O2c_ defect, leaving the excess charge to form polarons and instead adsorb at hollow sites, which is in agreement with our experimental STM observations where they quickly sinter into clusters with increasing temperature at low coverage.

The interaction between adsorbates and polarons is further complicated by the presence of additional species which could alter the surface charge balance. In this respect, it is also important to note that the TiO_2_(110) surface is fully oxidized under realistic conditions. Therefore, V_O_ is repaired by H_2_O or O_2_ molecules, leading to hydroxylated TiO_2_(110) or oxidized TiO_2_(110) surfaces. The oxidized TiO_2_(110) surface was suggested to bind Au_1_ stronger than the V_O_ site on the reduced TiO_2_(110) surface [53]. Contrary to the Au case, only Pt clusters were observed on the oxidized TiO_2_(110) surface.

Nevertheless, the presence of small polarons is important even for a realistic TiO_2_(110) surface, because repair of an oxygen vacancy by H_2_O results in two electron polarons arising from two surface OH groups. Clearly, the modeling of such complicated multi-polaron configurational space (which might also involve the simultaneous formation of electron and hole polarons) is clearly unfeasible via conventional DFT. In order to efficiently explore the energy landscape, novel automated machine-learning based methods must be implemented and employed to determine the most favorable (structural and electronic) configurations and the most likely dynamical paths [74].

## Supplementary Information

Below is the link to the electronic supplementary material.

Supplementary material 1 (DOCX 2359.3 kb)
