# Supplementary material for: Role of Polarons in Single-Atom Catalysts: Case Study of Me1 [Au1, Pt1, and Rh1] on TiO2(110)
Source: Top Catal. 2022 Jun 27;65(17-18):1620–30. doi: 10.1007/s11244-022-01651-0 (PMC9668789; doi:10.1007/s11244-022-01651-0)
Supplement: Supplementary file 1 — Supplementary material 1 (DOCX 2359.3 kb) [file 11244_2022_1651_MOESM1_ESM.docx]

**Role of Polarons in Single-Atom Catalysts: Case Study of Me_1_ [Au_1_, Pt_1,_ and Rh_1_] on TiO_2_(110)**

Panukorn Sombut^1^, Lena Puntscher^1^, Marlene Atzmueller^1^, Zdenek Jakub^1^, Michele Reticcioli^2^, Matthias Meier^1,2^, Gareth S. Parkinson^1^ and Cesare Franchini^2,3*^

^1^Institute of Applied Physics, TU Wien, 1040 Vienna, Austria

^2^Faculty of Physics, Center for Computational Materials Science, University of Vienna, 1090 Vienna, Austria

^3^Alma Mater Studiorum, Università di Bologna, 40127 Bologna, Italy

* E-mail: [cesare.franchini@univie.ac.at](mailto:cesare.franchini@univie.ac.at)

**SUPPORTING INFORMATION**

**DFT+U calculations**

**
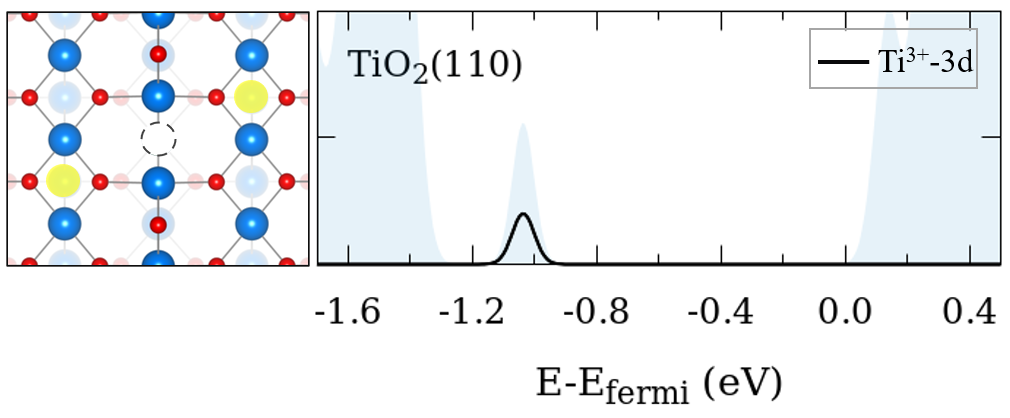
**

**Fig. S1** The clean TiO_2_(110) surface with an oxygen vacancy and the most stable polaronic configuration with its DOS and pDOS of Ti^3+^(3d, polarons) are filled with light blue and black line, where O, Ti^4+^ and Ti^3+^(polaron) are small red, big blue and big yellow spheres, and V_O_ is a dashed-circle, respectively.


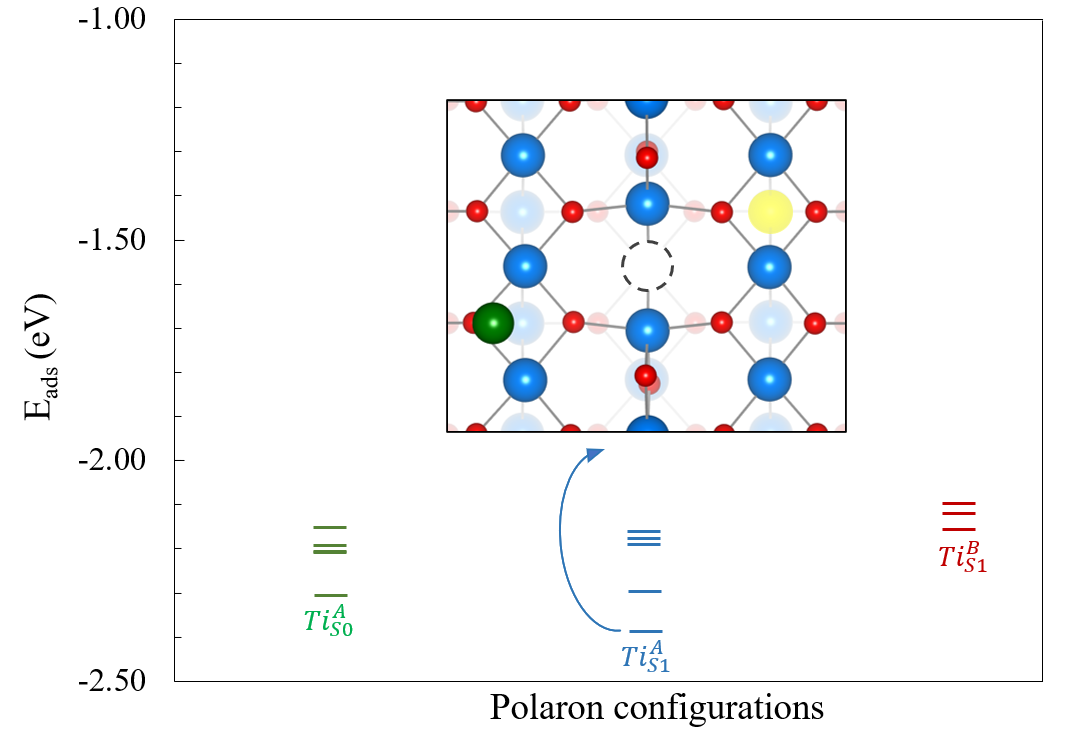


**Figure S2**: Adsorption energy (E_ads_) of Pt^−^ adsorbed on top of O_3C_ atom with one polaron remaining on the TiO_2_(110) surface for which different polaron configurations have been investigated. Each adsorption energy corresponds to a local minimum with a fixed Ti^3+^(polaron) localization on the TiO_2_(110) surface at S0 and S1 layers, where O, Ti^4+^, Ti^3+^(polaron), Pt_1,_ and V_O_ are red, blue, yellow, green, and dashed-circle, respectively.


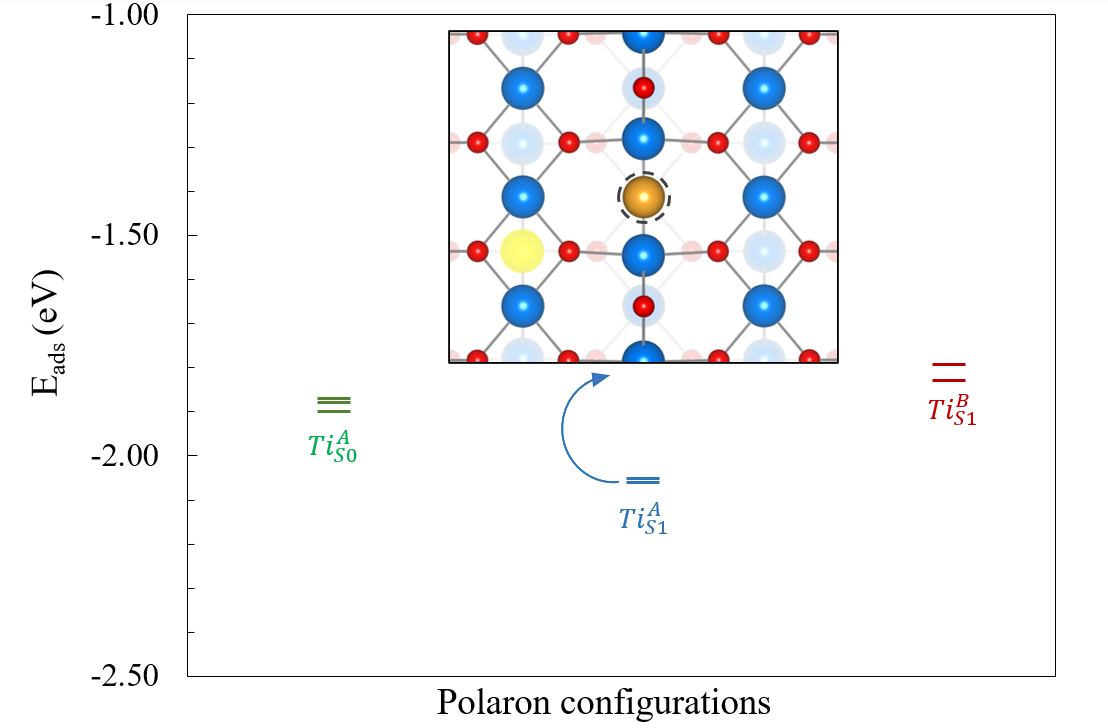


**Figure S3:** Adsorption energy (E_ads_) of Au^−^ adsorbed at V_O2C_ with one polaron remaining on the TiO_2_(110) surface for which different polaron configurations have been investigated. Each adsorption energy corresponds to a local minimum with a fixed Ti^3+^(polaron) localization on the TiO_2_(110) surface at S0 and S1 layers, where O, Ti^4+^, Ti^3+^(polaron), Au_1,_ and V_O_ are red, blue, yellow, yellow-brown, and dashed-circle, respectively.


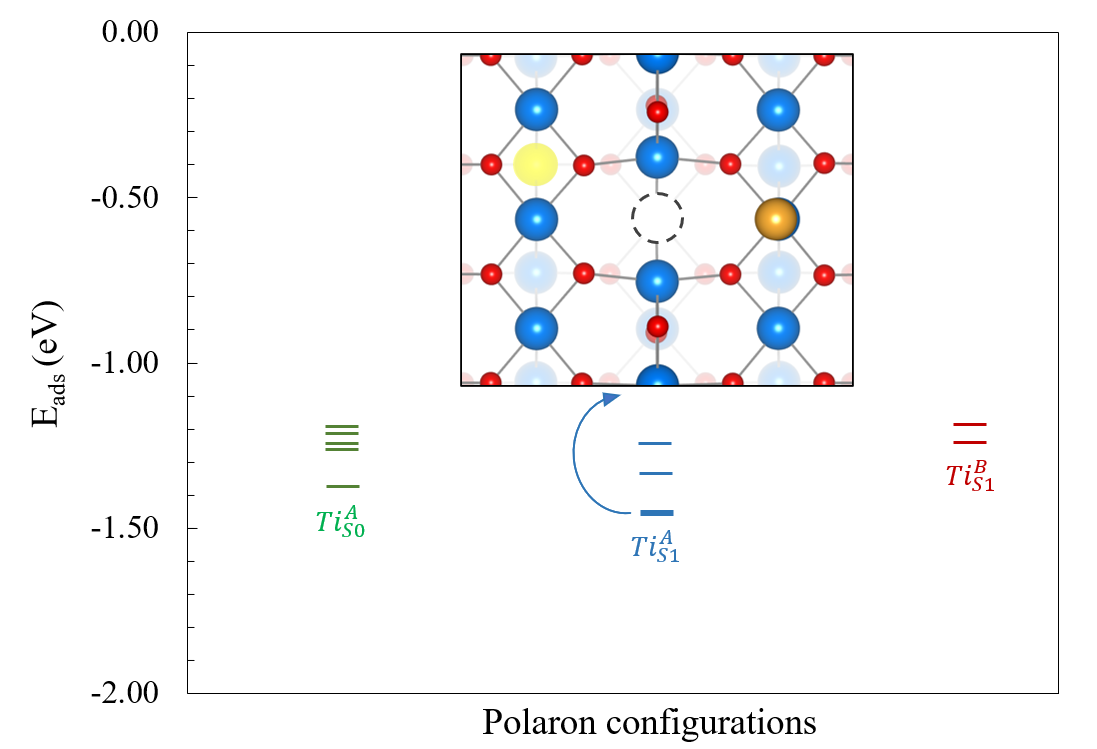


**Figure S4**: Adsorption energy (E_ads_) of Au^−^ adsorbed on top of Ti_5c_ atom with one polaron remaining on the TiO_2_(110) surface for which different polaron configurations have been investigated. Each adsorption energy corresponds to a local minimum with a fixed Ti^3+^(polaron) localization on the TiO_2_(110) surface at S0 and S1 layers, where O, Ti^4+^, Ti^3+^(polaron), Au_1,_ and V_O_ are red, blue, yellow, yellow-brown, and dashed-circle, respectively.


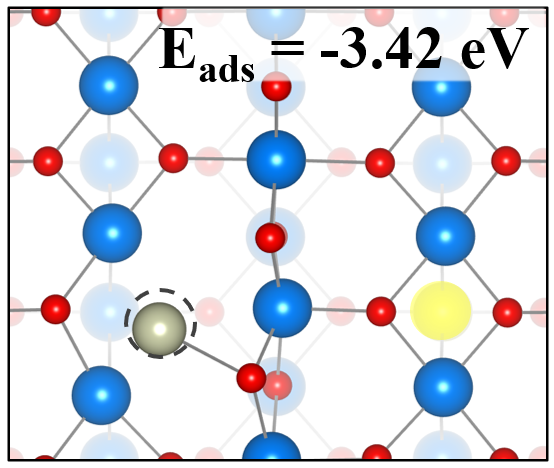


**Figure S5**: Rh_1_ adsorbed at V_O3c_ is a thermodynamically stable adsorption site (E_ads_ = −4.82 eV) where O, Ti^4+^, Ti^3+^(polaron), Rh_1,_ and V_O_ are red, blue, yellow, gray, and dashed-circle, respectively.


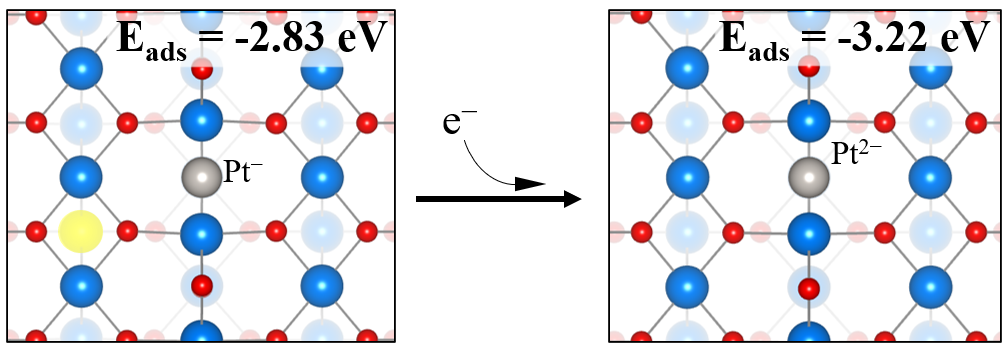


**Figure S6**: Adsorption energy (E_ads_) of Pt^−^ and Pt^2−^ at V_O2c_. Charge transfer occurring at V_O_ site modifies the stabily by 0.39 eV.

**RT STM images of Pt_1_ on the TiO2(110) surface**


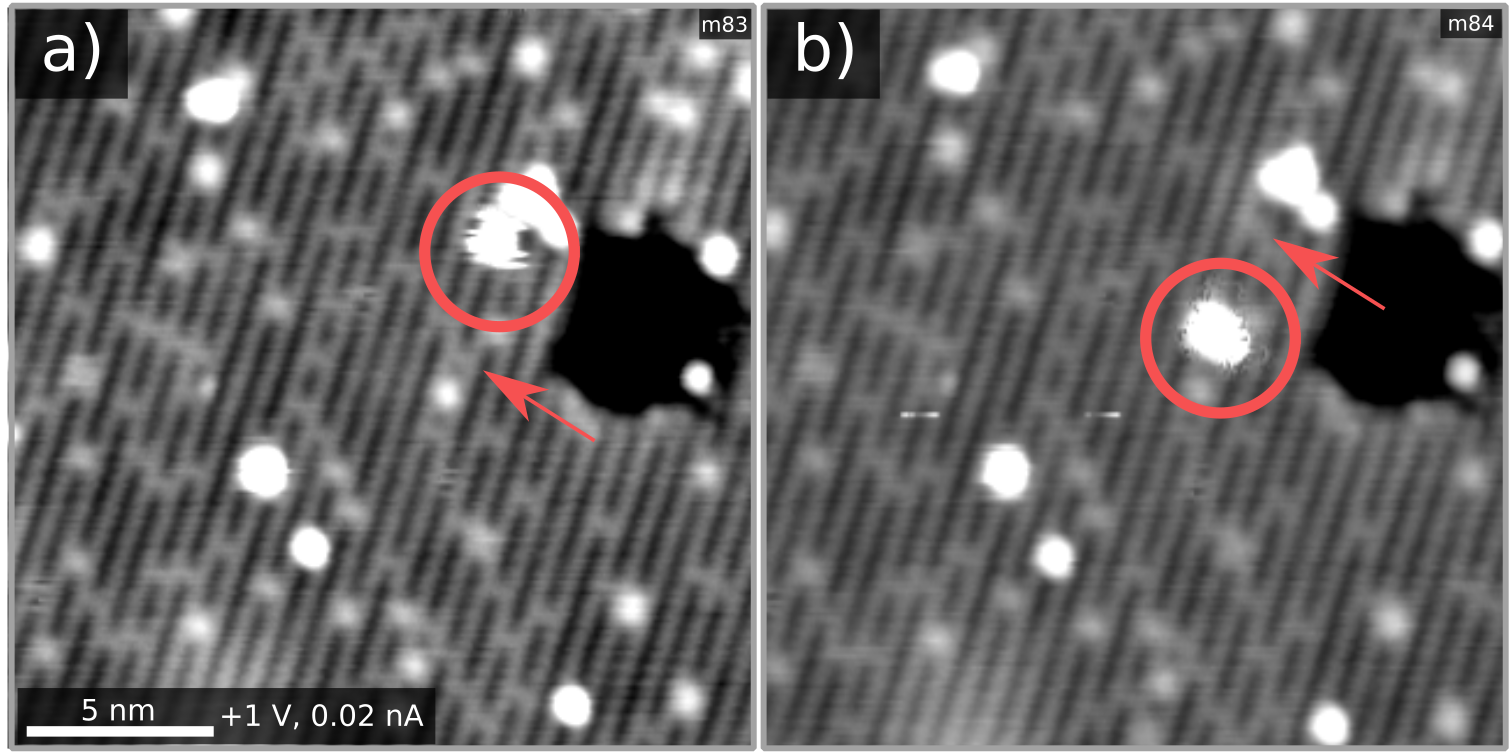


**Figure S7:**  RT STM images of Pt_1_ adatoms, showing the hopping from one O vacancy to another (marked in red circle).


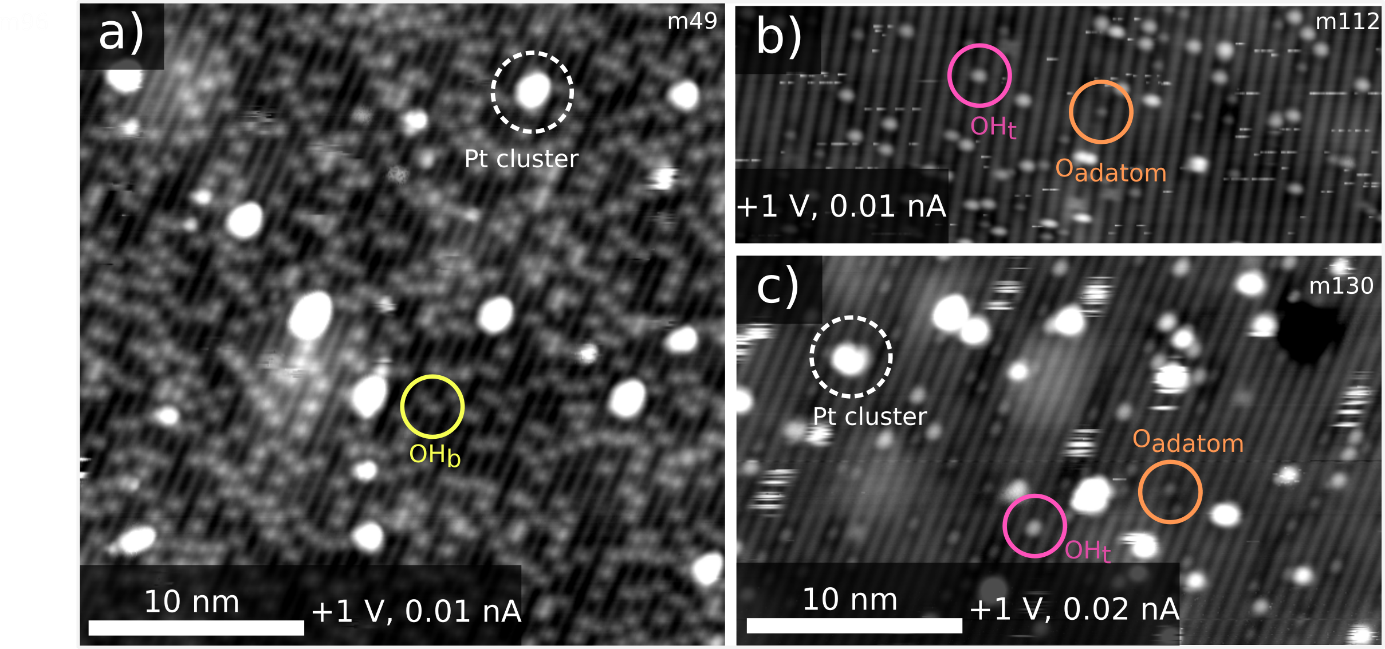


**Figure S8:** RT STM images 0.01 ML Pt on a) a hydroxylated surface and c) on an oxidized surface. Only Pt cluster (white dashed) can be seen. b) shows the oxidized surface before the Pt deposition. Oxygen adatoms and hydroxyl groups adsorb on the 5-fold Ti atoms.

O_2_ splits at the 2-fold oxygen vacancies. One atom heals the vacancy and the other one adsorbs on the 5-fold Ti (orange). Due to background water, some of the vacancies were already hydroxylated before the oxidation. They react with the oxygen adatoms, which also heals the vacancy and forms so called “terminated OH” which adsorb at the 5-fold Ti (pink) [1]

**References**

1. Du Y, Deskins NA, Zhang Z, et al (2009) Imaging consecutive steps of O 2 reaction with hydroxylated TiO 2(110): Identification of HO 2 and terminal OH intermediates. J Phys Chem C 113:666–671. https://doi.org/10.1021/jp807030n
